# Potential of Commercial Wood-Based Materials as PCB Substrate

**DOI:** 10.3390/ma15072679

**Published:** 2022-04-05

**Authors:** Kirsi Immonen, Johanna Lyytikäinen, Janne Keränen, Kim Eiroma, Mika Suhonen, Minna Vikman, Ville Leminen, Marja Välimäki, Liisa Hakola

**Affiliations:** 1 Sustainable Products and Materials, VTT Technical Research Centre of Finland, 02044 Espoo, Finland; janne.keranen@vtt.fi (J.K.); minna.vikman@vtt.fi (M.V.); 2 Packaging Technology, Lappeenranta-Lahti University of Technology LUT, 53850 Lappeenranta, Finland; johanna.lyytikainen@lut.fi (J.L.); ville.leminen@lut.fi (V.L.); 3 Digital Technologies, VTT Technical Research Centre of Finland, 02044 Espoo, Finland; kim.eiroma@vtt.fi (K.E.); mika.suhonen@vtt.fi (M.S.); marja.valimaki@vtt.fi (M.V.); liisa.hakola@vtt.fi (L.H.)

**Keywords:** cellulose, veneer, printed circuit board, fire retardant, screen printing, biodegradation

## Abstract

In our research on sustainable solutions for printed electronics, we are moving towards renewable materials in applications, which can be very challenging from the performance perspective, such as printed circuit boards (PCB). In this article, we examine the potential suitability of wood-based materials, such as cardboard and veneer, as substrate materials for biodegradable solutions instead of the commonly used glass-fiber reinforced epoxy. Our substrate materials were coated with fire retardant materials for improved fire resistance and screen printed with conductive silver ink. The print quality, electrical conductivity, fire performance and biodegradation were evaluated. It was concluded that if the PCB application allows manufacturing using screen printing instead of an etching process, there is the potential for these materials to act as substrates in, e.g., environmental analytics applications.

## 1. Introduction

The EU’s Industrial strategy [1] aims for scalability, setting of global standards, and building a more circular economy based on digital and green capacities. Specific pillars for a future green electronics industry include lower power consumption, renewable materials, low quantities of raw materials, a decrease in resources, including energy, and other eco-design opportunities [2]. Eco-design strives for, e.g., use of materials with less environmental impact, use of fewer materials in manufacturing, use of fewer resources during manufacturing, and the generation of less pollution and waste [3]. As a subtractive method, traditional etching-based processes have difficulties in meeting these goals, given their use of hazardous chemicals, generation of material waste, and poor compatibility with bio-based materials. Compatibility issues with bio-based materials for chemical etching are related to process impurities from dissolved substrate, poor tolerance to the high temperatures used during processing, and high porosities, leading to excessive conductive material absorption and penetration, the latter specifically with cellulose and paper-based materials. Printing-based additive manufacturing, on the other hand, has the capability to meet eco-design requirements in terms of lower energy consumption [4,5], and compatibility with bio-based materials.

Conventional electronic devices are primarily fabricated from polymeric and composite substrates, such as polyimide and FR4, respectively. The electronics industry can specifically decrease its environmental impact by shifting from fossil-based materials to bio-based materials, decreasing its use of metals and utilizing printing-based additive manufacturing processes [4]. It is estimated that additive manufacturing processes, such as printing, powered by electricity generated from renewable energy, use one-tenth of the materials of traditional factory production, resulting in a clear reduction in CO_2_ emissions and use of the earth’s resources [5]. By employing additive methods, energy consumption during manufacturing can be even five times less than with conventional methods.

Flexible printed circuits offer several advantages compared to rigid circuits, including reduced package dimensions, reduced weight, and optimization of component real estate, as well as the use of printing-based manufacturing. Although printed electronics is a sustainability opportunity, its main environmental challenges at the moment come from the use of fossil-based substrate materials and metal conductors [6,7]. Currently printed electronics is based mostly on polymeric substrates, such as polyethylene terephthalate (PET). New materials originating from renewable and bio-based resources should also be considered for printed electronics.

Sustainable and printable substrate materials can be found among different cellulose and wood-based materials, bioplastics, and biocomposites [8,9,10,11,12,13]. Electrochemical deposition of copper and tin has been demonstrated on poly(lactic acid) (PLA) substrate and vapor phase reflow soldering has been performed on PLA and cellulose acetate substrates based on copper laminates [14,15]. Wood-based substrate materials are one opportunity for printed circuit board (PCB) manufacturing to replace current plastic and composite materials [16]. Wood-based materials possess advantages, such as low cost, flexibility, biodegradability, compostability, and ease of disposal through fiber recycling or incineration. Biodegradability is an important aspect in cases where electronics end up in biological environments at some point of their life cycle, such as sensors for environmental condition monitoring or for precision agriculture. A wood-based electronic application is demonstrated, for example, by the coating of veneers with Cu-nanowires [17].

The use of PCBs in electronic devices usually sets specific requirements for surface roughness and adhesion, mechanical durability, temperature stability and moisture tolerance; the use of fire retardants is also often expected [14,15,18,19]. The capability of wood-based materials to meet these expectations might be limited, but the work reported in this paper aims to show that there are potential material alternatives already available which are compatible with electronic performance and printability requirements.

The aim of this work was to determine if there is any possibility for wood-based materials, such as cardboard or veneer, to act as substrate materials for printed electronics and especially focus on PCB manufacturing by screen printing. Due to digitalization and the growth of the IoT market, new electronic solutions are being introduced, and the need for sustainable solutions is critical to many sectors where electronics will be embedded or released to biological environments at some point of their lifecycle. Therefore, the biodegradability of electronics is important, and since the substrate constitutes a major part of any device, changing it from a composite or plastic to a biodegradable option is a desirable step.

## 2. Materials and Methods

### 2.1. Materials

The substrate materials selected for this study were PankaMax cardboard (CB) from Pankaboard, Pankakoski, Finland (thickness of 1.75 mm and area density of 650 g/m^2^) and KoskiPly Birch Exterior AB/B (1.5 mm thick veneer) from Koskisen Oy, Kärkölä, Finland.

The fire-retardant materials selected for use were FireProof P2 and FireProof W1 from Kiilto Oy (Kiilto Oy, Lempäälä, Finland), Organowood^®^ (Organowood AB, Täby, Sweden) Protection 01 from OrganoWood AB and Zeopol^®^ 33 from Evonik (Evonik Resource Efficiency GmbH, Hanau-Wolfgang; Germany). FireProof P2 (FPP2) is an environmentally safe fire retardant designed for paper and cardboard applications. It is a liquid suitable for spray spreading with a density of 1.25 g/m^3^, a pH of 13, and dry material content of 27%. It should meet fire class E (SFS-EN ISO 11925) if applied at 10 g/m^2^ on cardboard. FireProof W1 (FPW1) is a fire retardant, which is specifically developed for treatment of spruce veneer, using spray or brush coating. It has a pH between 5–7 and a DIN 4 viscosity of 13 ± 2 s. It is specified to meet Euroclass B-s1 d0 when applied (on spruce veneer) at 250 g/m^2^. OrganoWood^®^ Protection 01 (OW01) is a water-based wood treatment product composed of silicon minerals and natural plant compounds. It is intended for use in the surface treatment of wooden products, such as solid wood or particleboards. It is a transparent liquid with a density between 1.2–1.4 g/m^3^ and a pH of 10–11. When used for spruce panel coating, it meets reaction to fire classification Cs1 d0, according to EN13501-1 as dipped on surface at 0.33 L/m^2^ (about 250 g/m^2^). Zeopol^®^ 33 (Z33) is a clear low viscous sodium silicate solution with 37% dry material content, a density of 1.378 g/m^3^ and pH of 11. It is suitable for spray spreading and is widely used in pulping or the recycled paper industry.

The ink used for the screen-printing trials was Asahi LS-411AW microparticle silver paste from Asahi Chemical Research Laboratory. It is a one-component, polymer-type silver paste with a polyester binder. It has a solid content of 75–78% and a viscosity of 200–300 ps at 25 °C. The ink is cured at 150 °C for 20 min. The typical sheet resistance for a 10 µm layer is <40 mΩ/sq.

### 2.2. Coating with Fire Retardants

Fire-retardant coatings were applied using the spray method on one side of the substrates. The application amounts are displayed in Table 1. Of the fire-retardants used, OW01 was the easiest to apply, using a spray. Spraying was performed in a fume hood and drying was done in an oven with an exhaust exit to outside air. The application of Z33 using the spray method was quite similar to that of OW01, but first needed dilution from the original dry material content of 20% to 10% using purified water. The application of FPP2 required that some practical additional protection measures were taken in addition to the earlier protection measures of gloves, goggles, and fume hood. The fume hood was protected with a plastic film for easier cleaning, and a protective suit (Tyvek PRO-TECH F TOPGUARD) (Indutex GmbH, Krefeld, Germany) and gas mask (A2B2E2K2-P3 R filter) (3M, Cummins’ Neillsville, Wisconsin, USA) were also used. Any waste created were handled as hazardous waste. The same measures were followed for application of FPW1 as well, as it can corrode the metal of the fume hood.

### 2.3. Analytics for Surface Characteristics

A Theta optical tensiometer (Biolin Scientific, Gothenburg, Sweden) was used for measuring the contact angle of water on the uncoated and coated surfaces. Samples were conditioned and measured at 23 °C and 50% relative humidity. Measurements were performed with deionized water at a drop volume of 3 µL. The reported contact angle values are average values from five measurements.

The surface roughness of the uncoated and coated samples was measured from a 15 × 15 mm area using a Keyence VR-3200 optical 3D-profilometer (Keyence, Itasca, Illinois, USA). The reported roughness values are the roughness averages of the measured areas.

### 2.4. Limiting Oxygen Index (LOI)

The flame resistance of the fire-retardant-coated substrates was evaluated by the limiting oxygen index method (LOI), according to ISO 4589-2:2017. The standard specified methods for determining the minimum volume fraction of oxygen, in an admixture with nitrogen that will support combustion of small vertical test specimens under specified test conditions. The results are defined as oxygen index (OI) values. Samples were cut from coated cardboard or veneers into 2-cm-wide and 20-cm-long self-standing samples. The samples are placed vertically on the sample holder, and the oxygen content is adjusted. The measurement is made by increasing the relative oxygen amount in steps of 0.5% compared to the nitrogen, igniting the samples from the top, and analyzing if the material catches fire and how long it will burn, both time wise and length wise. The result is when the material can sustain burning for 3 min or the burning length is 5 cm in under 3 min time. Typically, 0.5% differences show differences in material burning properties. In very homogeneous samples, even a smaller step can show a big difference. The result is based on four to six measurements.

### 2.5. Screen Printing

The printing tests were performed using an EKRA E2 sheet-based screen printer and Asahi LS-411AW microparticle silver paste. A 325-24 (mesh count (LPI), wire diameter (µm)) screen mesh, squeegee hardness and angle of 65 ShA/45°, and printing speed of 40 mm/s were used. The printed samples were dried with hot air in a box oven at 150 °C for 20 min. The layout consisted of a conductor line and line space patterns with varying width (100, 150, 200, 300, 500, and 1000 µm) and orientation, as well as larger (1.5 × 1.5 cm) rectangular areas.

### 2.6. Analytics for Printed Patterns

The resistance (R) of printed samples with a size of 15 × 15 mm square was measured using a digital multimeter Keithley 2700 (Keithley Instruments LLC, Solon, Ohio, USA) connected to a four-point probe setup with probing tips arranged linearly with an equal 600 µm spacing. The equation for an infinite sheet, where the thickness of the layer is much smaller than the probe spacing, was then used for calculating the sheet resistance

Rs (Ω/sq.) = πR/ln(2)

For the line samples, the resistance (R) was measured using a digital multimeter (Keithley 2700). Using the width (w) and length (l) of the line, the following equation for calculating the sheet resistance (Rs) was applied:

Rs (Ω/sq.) = Rw/l

An optical microscope was used to study the print quality attributes of the printed line space patterns. The number of measurements for the square patterns was ten and a maximum of five for the lines, due to a limited number of uniform printed lines in samples.

### 2.7. SEM

The morphology of samples was studied using a Scanning Electron Microscope (SEM) JEOL JSM T100 (JEOL Ltd., Tokyo, Japan) with a voltage of 10 kV. Images were taken from the cross-cut surface of samples containing a silver printed pattern.

### 2.8. Biodegradation in Soil

The biodegradability of materials was evaluated using ISO 17665:2012, which is based on CO_2_ evolution. Instead of passing carbon-dioxide-free air over the soil, and then determining the carbon dioxide content of the air as described in the standard, CO_2_ concentrations were analyzed from the head space volumes without passing air over soil. 

Soil was collected from an agricultural field located in Helsinki, Finland. The pH of the soil was 6.4, organic matter content was 9% (dry weight) and the C/N ratio was 19. The soil was sieved to a 2 mm particle size, and the final soil moisture content was adjusted to 76% (~30% of the water holding capacity). For each replicate, 500 mg of material was mixed with 75 g of soil (dry weight) in a 1000 mL glass bottle sealed with septum and screw cap with a hole. Three replicates were prepared for each tested material, for blank and for microcrystalline cellulose with particle size 20 µm (Sigma-Aldrich/ Merck KGaA, Darmstadt, Germany) that was used as a reference compound. Samples were milled using an IKA A11 analytical mill (IKA, Staufen, Germany) and sieved to a 500 µm particle size. The carbon content of the materials and the nitrogen and carbon content of the soil were evaluated using a Flash 2000 EA CHNS-O (Thermo Fisher Scientific Oy, Espoo, Finland) elemental analyzer.

Carbon dioxide concentrations in the head space volumes of the bottles were measured at regular intervals by inserting a needle through the septum of the bottles and directly measuring the CO_2_ concentration using a Servoflex MiniFoodPack 5200 infrared analyzer (Servomex, Crowborough, UK). Bottles were aerated to remove excess CO_2_, closed with a septum and screw cap, and CO_2_ concentrations were measured for a second time. The net CO_2_ production from the test materials was calculated by subtracting the average amount of CO_2_ produced in the blank soils (no sample) from the amount of CO_2_ produced in the test material bottles. The biodegradation percentages were calculated from the ratio between the net CO_2_ production and the theoretical CO_2_ production calculated on the basis of the elemental carbon content of the material.

## 3. Results

### 3.1. Results for Substrate Properties

**Figure 1:**
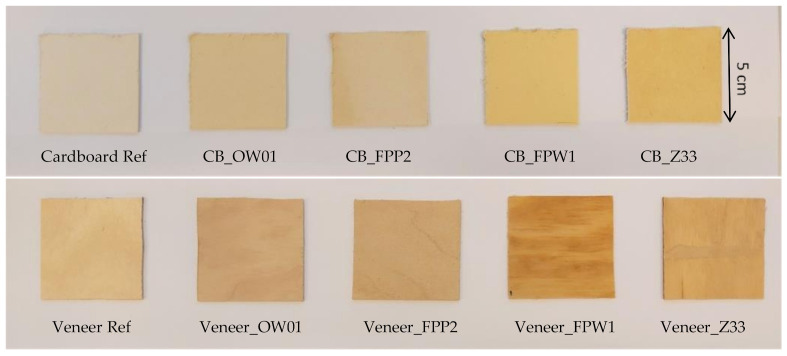
Images of fire-retardant-coated samples (coated side up). Sample size 5 × 5 cm.

Fire-retardant-coated cardboard and veneer samples are presented in Figure 1. Coating thicknesses and additional sample information are given in Table 1.

Fire retardants behaved quite differently on cardboard and veneer substrates. On the more porous cardboard the OW01 and Z33 retardants, which are water-based and contain silicon minerals, absorbed into the fiber structure but similar absorption did not occur on the veneer. FPP2, which is designed for paper and cardboard applications, gave very similar coatings on both the cardboard and veneer. FPW1, developed for veneer applications with thick layers (250 g/m^2^), provided the heaviest layer on the cardboard (143 g/m^2^) and veneer (86 g/m^2^).

Some challenges occurred in spray coating due to the different viscosities of the fire-retardant materials that led to differences in the final coating amounts. The different application behavior of the fire-retardants is seen by comparing the thicknesses and mass changes before and after the applications. If the thickness is not observed to increase, the applied amount is presumably absorbed into the structure. Such results indicate that the applied amounts can penetrate into porous structures. In practice, these retardants are seen as easy to apply using the spray method.

#### 3.1.1. Surface Properties

Contact angles of water and surface roughness were measured from uncoated and coated samples (Table 2). In general, the contact angle values were low for the coated surfaces indicating high wettability. In many cases, the contact angle value was not measurable due to complete spreading and/or absorption of the droplet on the surface.

Except for the FPP2 coating, the surface roughness increased notably on the coated cardboard. On the veneer, the change in roughness on the coated surface was smaller with decreases in roughness was also being observed. The smoother surface of the veneer substrate enabled more even when coating.

#### 3.1.2. LOI Results

**Figure 2:**
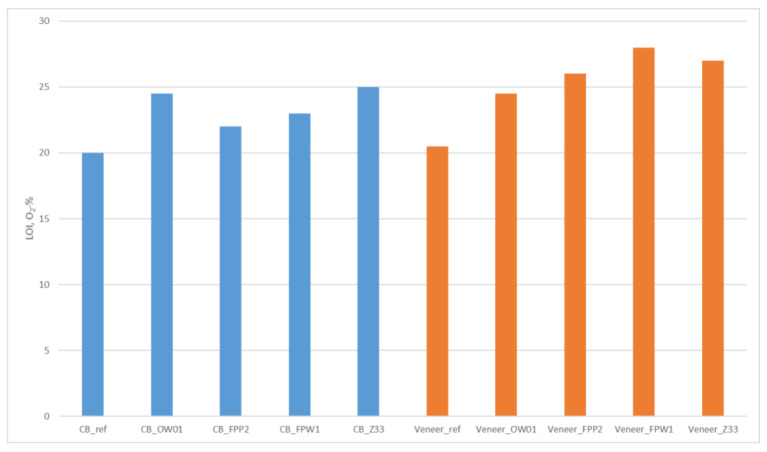
Limiting Oxygen Index (LOI) results for substrates.

LOI-results for the uncoated and coated substrates are presented in Figure 2 and show improved fire retardancy in all coated substrates. The best fire resistance for CB was achieved with the silicon-based OW01 and Z33 retardants for which LOI values of 24.5 and 25 O_2_-%, respectively, were determined. Both fire retardants were also partially absorbed by the cardboard structure. The best fire protection for veneer was achieved with the FPW1, which was specifically developed for veneer protection, and Z33 retardants, for which LOI values of 28 and 27 O_2_-%, respectively, were determined. 

### 3.2. Results for Screen Printed Samples

The results for sheet resistance and line quality for the screen-printed samples are summarized in Table 3. The sheet resistance over areas of square (15 × 15 mm^2^) and line (0.5 × 18 mm^2^) patterns was measured. For the cardboard samples, the square pattern areas yielded significantly lower sheet resistance values than the line patterns. These results are likely due to the large contribution of the poor print quality of the line patterns to the measured resistance. For the larger area square patterns, the continuous layer of printed silver is sufficient to yield reasonable resistance values. For the veneer samples, the discontinuities in the printed layer, resulting from the ink penetrating into specific areas of the wood grain structure, yielded higher resistance values.

Print quality was quantified by measuring the actual line width of nominally 500 µm wide lines, and by determining the minimum nominal gap width between parallel lines. Ink spreading was significant on the rough and porous cardboard substrate, and as a result, none of the gaps in the printing layout remained open. For the veneer, the line width remained close to nominal. The minimum gap width was the lowest for the untreated reference sample (Veneer_ref). The fire-retardant coating improved the printability of the veneer to the extent that no discontinuous lines were observed.

**Figure 3:**
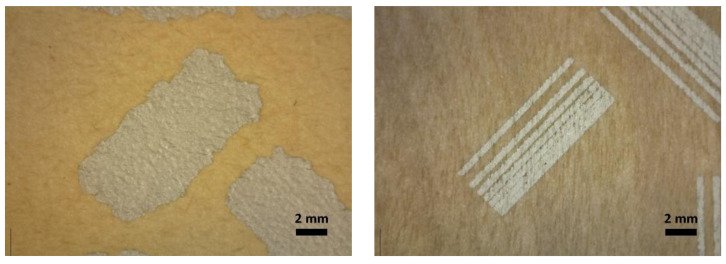
Minimum-gap-width test structure printed on CB_ref (**left**) and Veneer_ref (**right**) substrates.

**Figure 4:**
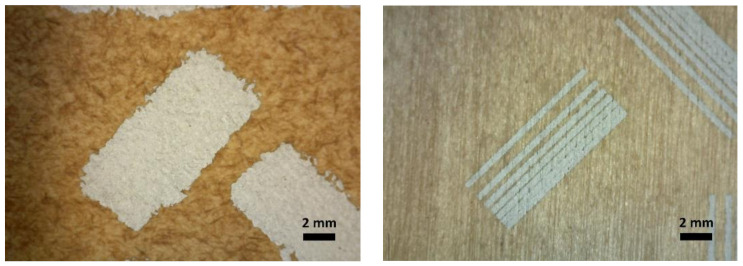
Minimum-gap-width test structure printed on CB_Z33 (**left**) and Veneer_Z33 (**right**) substrates.

The morphology and a closer look at the silver printed CB_ref and veneer_ref. samples and with samples containing Z33, are presented in Figure 3 and Figure 4.

The images in Figure 3 and Figure 4 are representative of the print quality of the screen-printed silver ink on untreated and treated cardboard and veneer substrates. All images are taken from the minimal gap width test structure.

For the cardboard substrates, the fire-retardant coatings slightly increased the spreading of the ink and the sheet resistance of the printed lines. For the veneer substrates, line width results suggest that the fire-retardant coatings did not significantly affect the printing quality, whereas the minimum-gap results indicate that the fire-retardant coatings slightly increased the spreading of the ink. The main effect seems to be that the fire-retardant coatings closed the veneer surface to a degree that discontinuities in the conductor were not observed. For both substrates, the lower contact angles of the treated substrates compared to that of the untreated substrates (Table 3) is in line with the observed increased spreading of the ink in the minimum-gap width structures for the treated surfaces. For improved printing and electrical performance, a primer coating is suggested for both substrates to decrease the porosity and roughness of the surfaces.

**Figure 5:**
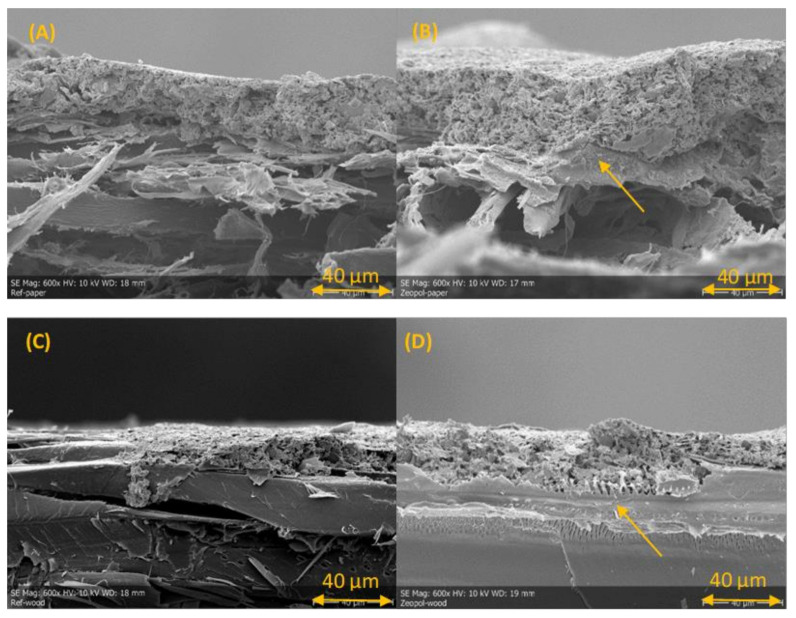
SEM images of cross-cut samples of CB_ref. (**A**), CB_Z33 (**B**), veneer_ref. (**C**) and veneer_Z33 (**D**) with printed silver on the surface. Arrows in images (**B**,**D**) show the location of the Z33 layer. Image C shows the edge of the printed silver pattern.

The SEM images in Figure 5 show the printed silver layer on top of the substrates and prove the silver layer thickness of 20 to 50 µm. Images A and B for the CB also show the high porosity of the paper substrate compared to the veneer in images C and D. Image B, containing the FR Z33 on CB, shows the potential unevenness of the FR layer and even partial absorption in the CB structure. Z33 forms a more even layer on veneer surface that can be seen in image D, pointed by the arrow. The thickness of the Z33 coating on veneer is uneven, but at the same level as presented in Table 3, 47 µm. Image C shows the edge of the silver print on veneer_ref and prove it to be quite sharp, which was also seen in Figure 4.

### 3.3. Results for Biodegradation Properties

**Figure 6:**
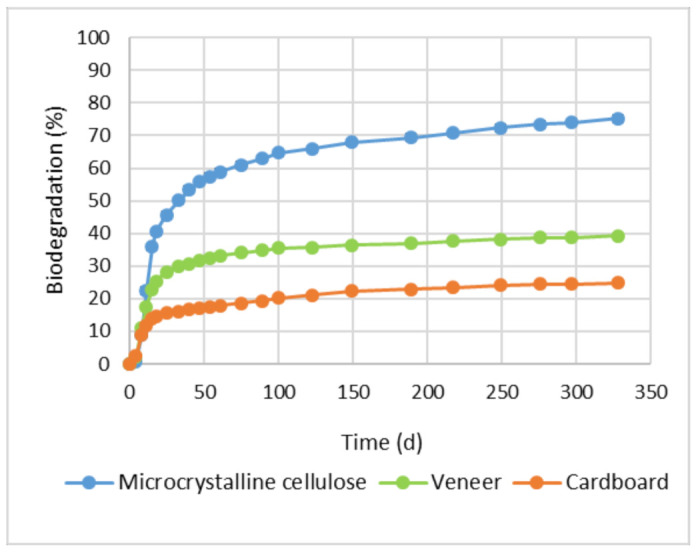
Biodegradation of uncoated veneer and cardboard in soil conditions. Biodegradation is calculated as ratio of measured CO_2_ and theoretically produced CO_2_.

As shown in Figure 6, the biodegradability (after 328 days of incubation) of uncoated veneer and cardboard was 39% and 25%, respectively. There are no threshold values available for the biodegradability in soil for these types of products. However, the EN 17033:2018 threshold value for biodegradability of mulching films during the maximum test period of 2 years is set at 90% as relative to a reference microcrystalline cellulose material. The relative biodegradability of veneer and cardboard using microcrystalline cellulose as a reference is calculated to be 52% and 33%, respectively.

The typical testing time in the ISO 17,665 soil biodegradation test is 6 months, but the test can be continued for up to 2 years until a plateau is reached. After one year (more specifically 328 days, due to the limited timeline in the project) of incubation, carbon dioxide evolution from the samples is still higher compared to background (soil without samples), and consequently higher biodegradability results would be expected if the tests were continued up to two years.

After 328 days of incubation, the biodegradation of the reference microcrystalline cellulose compound was 75%. According to ISO 17665:2012, the test is valid if the degree of biodegradation of the reference material is more than 6 % at the end of the test period.

## 4. Discussion

The aim of this work was to analyze if there is any possibility for wood-based materials, such as cardboard or veneer, to act as substrate materials for printed electronics, focusing on printed circuit boards (PCB) manufactured by screen printing. Potential application areas for wood-based PCBs would be, for example, in environmental monitoring where biodegradability is needed. An extra-bulky, uncoated cardboard (CB) and 1.5 mm thick birch veneer were selected as base materials. To provide some level of fire resistance for the selected substrates, and to provide smoother surfaces for screen-printing, the substrates were coated with fire retardants. 

Four different fire-retardant (FR) materials were selected for testing and were spray coated on the substrates without adjusting the viscosity to the same level. OW01 and Z33 are silicon containing water-based materials, FPP2 is a commercial FR material designed for cardboard applications and FPW1 is a commercial FR material developed for spruce veneer. The application varied on CB from 17 to 143 g/m^2^. OW01 was applied at the lowest level, and it is specified to attain C classification when applied at 250 g/m^2^ using dip-coating. However, the 17 g/m^2^ amount, when absorbed on the cardboard fiber structure, was able to provide some protection as indicated by the LOI value of 24.5 O_2_-%. Z33 was absorbed onto the fiber structure at 37 g/m^2^ and gave the best fire resistance (LOI value of 25 O_2_-%). FPP2 should meet fire class E in the amount of 10 g/m^2^. Here it was applied at 44 g/m^2^ on the cardboard surface and provided a LOI value of 22 O_2_-%. Exceeding an LOI value of 21 O_2_-% indicates that the material requires a higher oxygen content to support combustion than is provided under normal conditions. 

The applied FR amounts on veneer varied from 34 g/m^2^ (OW01) to 84 g/m^2^ (FPW1). The best fire protection was attained with FPW1 at an LOI value of 28 O_2_-%, which is a very good results as it is suggested to be used in the amounts of 250 g/m^2^ on veneer to meet the fire class C. Z33 was nearly as effective providing an LOI of 27 O_2_-% when applied at only 48 g/m^2^. It must be noted that LOI gives only an indication of material flammability resistance and does not confer any classification compliance. However, it does appear that Z33- or FPW1-coated veneer have the potential to provide some fire resistance for PCB solutions against electrical short circuits potentially causing ignition.

All the tested coatings gave higher roughness values and a lower contact angle, providing the surface with increased hydrophilicity for the carboard. The higher roughness and lower contact angle properties affected screen printing as wider line widths were observed compared to neat cardboard. The effects of these properties also resulted in higher sheet resistance values for the coated cardboards especially for 0.5 × 18 mm line pattern areas (see Table 3). The sheet resistance over the 15 × 15 mm squares for the cardboard samples ranges from 36 to 51 mΩ/sq, which is close to the optimal sheet resistivity level given for the silver ink used in screen printing (<40 mΩ/sq for 10 µm line thickness). The thickness of printed patterns is in the level of 20 to 50 µm and presented in Figure 5. Figure 3 and Figure 4 show that the lines are spread on the cardboard surface with no visible gap between them when printed close to each other. The roughness of the uncoated veneer was 6.6 µm being clearly smoother than that of the uncoated cardboard (22.6 µm).

The application of the OW01 and FPW1 FR coatings resulted in smoother surfaces that that of the untreated veneer as indicated by the roughness values of 6.0 µm and 1.2 µm, respectively. The roughness of coated veneer was 7.4 µm with Z33 and 10.2 µm with FPP2. The contact angle of veneer dropped significantly (from 103°) with all the FR coatings indicating a higher levelling of water and probably an improved levelling of printing inks on the surface. The characteristic of the veneer surface changed from hydrophobic when uncoated (>90°) to hydrophilic (<90°) after coating. The same change of hydrophobicity was also found for coated and uncoated cardboard. The line quality for the veneer samples was good, showing line widths of 400 (OW1) to 458 µm (uncoated) for a nominal screen-printed line width of 500 µm (see Table 3). The minimum gap width was smallest (150 µm) on the uncoated veneer which also featured the highest contact angle.

For the uncoated veneer, the sheet resistance over the 0.5 × 8 mm line pattern areas were unmeasurable due to line breaks and was 91 mΩ/sq over the 15 × 15 mm square areas. The best overall performance of the coated veneer samples was found with Z33 which provided sheet resistance of 71 mΩ/sq over the line pattern areas and 100 mΩ/sq over the square areas and also provided the best visual appearance for the printed patterns. A sheet resistance range of 300–600 mΩ for Al-coated veneer was reported by Wang et al. [20]. Li et al. coated veneer by sputtering copper on the surface at different temperatures and observed increases in sheet resistance from 40 mΩ up to >300 mΩ when the application temperature was raised from room temperature to 200 °C. It was assumed that, at higher temperatures, these increases may be caused by a reduction of the copper layer, due to a diffusion of ink on the surface or increases of surface roughness at an elevated temperature from the destruction of sealing strata on wood veneer [21]. The silver ink curing used in the work reported here was 20 min at 150 °C, and it is possible that similar effects on the veneer, which probably contains lignin and hemicellulose, may occur under the FR-coating. Unfortunately, surface properties were not analyzed after printing, but could be done in further studies. A similar increase in sheet resistance was not found for the FR-coated dense cardboard over the square areas.

If printed electronics are used in applications which end up in a natural environment, biodegradability is a desired property. Potential applications include, for example, sensors for precision agriculture when used in multitude, and collection after use is not feasible, and sensors for monitoring environmental conditions, such as weather with remote connection. Biodegradability is also important when considering the concept of single-use electronics, such as disposable health monitoring test devices, e.g., digital pregnancy tests ending up in domestic waste. Another example is electronic devices attached to compostable products, such as cellulose-based packages. In all of these cases, the electronics end up in a biological environment after use or is not collected properly for recycling, and creation of a new source of electronic waste should be avoided.

In this study, biodegradation of uncoated veneer and cardboard, used as a substrate material, was evaluated in a soil environment. Compared to a microcrystalline cellulose reference compound, the relative biodegradability of veneer and cardboard in the soil was 52% and 33%, respectively. There are no requirements available for the biodegradability of printed electronics in soil, but ISO 17,665 sets requirements for biodegradable mulching films used in agriculture. According to the standard, the relative biodegradation should be 90% of reference material in order to be considered as biodegradable.

One explanation for the low biodegradability values is that both veneer and cardboard contain considerable amounts of lignin. PankaMax cardboard is manufactured from groundwood pulp typically containing 27% of lignin. Veneer is composed of thin slices of wood bound together by glue. Lignin is known to reduce the biodegradability of cellulosic materials relative to complete mineralization and CO_2_ evolution [22,23]. It has also been demonstrated that a substantial fraction of ^14^C-labelled synthetic lignin was incorporated into humic compounds or other insolubles during composting, and only a minor part of the lignin was mineralized to carbon dioxide [24]. Similar behavior was observed in soil as disintegration of paper mulches in agricultural soil was reduced with increasing lignin content [25]. The formation of lignin is beneficial for soil but is difficult to take into consideration when the biodegradability of lignin-containing samples is evaluated.

In addition to lignin, other compounds and additives in veneer and cardboard can influence their biodegradability. The detailed composition of studied materials is not known, but a typical binder used in veneer is phenol formaldehyde, which has limited biodegradability [26]. When materials for PCB substrates are selected, the biodegradability of all material components, including coatings, as well as their environmental safety, should be considered.

Biodegradability is a complex topic, and merely employing biodegradable substrates is not sufficient for producing biodegradable electronics. There are numerous other printed and assembled components and circuitry required for achieving sufficient electrical performance, and biodegradability of these aspects should be studied in the future.

## 5. Conclusions

In this study, the potential for wood-based materials such as very dense cardboard and veneer to act as substrate material for printed electronics, especially in PCB manufacturing by screen printing, was examined. Some fire resistance is typically needed for PCBs to avoid ignition due to electrical shortcuts, which is why four different fire-retardant (FR) coatings were applied on the substrates by spraying. The FR coatings resulted in significant changes to both the cardboard and veneer surfaces changing them from hydrophobic to hydrophilic. Microparticle silver paste was printed on samples using screen printing and the sheet resistance was at an optimal level (40 mΩ/sq) for cardboard samples over larger areas (15 × 15 mm), but the print quality was better on veneer samples with, sheet resistances over 100 mΩ/sq. These substrates may find application in environmental or medical analytics where biodegradation can be an issue, and biodegradability of these substrates were analyzed and verified as well. It can be concluded that, from a screen-printing point of view, veneer has a greater potential to act as a PCB substrate when coated with sustainable, non-toxic fire FR material than cardboard. However, more research is needed on substrate surface treatments and application-focused design related to the replacement of etching processes with screen printing before actual wood-based PCB products can be available.

## Figures and Tables

**Table 1:** Thickness, weight and density of uncoated and fire-retardant-coated cardboard (CB) and veneer samples.

Sample	Grammage (g/m^2^)	Thickness (µm)	Coating Thickness, (µm)	Coating Grammage (g/m^2^)	Density, (kg/m^3^)
CB_ref	596 ± 2	1544 ± 10	0	0	386.1 ± 2.2
CB_OW01	613 ± 1	1528 ± 11	absorbed	17	401.1 ± 3.2
CB_FPP2	640 ± 13	1580 ± 22	36	44	404.7 ± 10.4
CB_FPW1	739 ± 6	1602 ± 26	58	143	461.4 ± 2.4
CB_Z33	633 ± 0	1540 ± 7	absorbed	37	411.0 ± 1.7
Veneer_ref	1149 ± 25	1449 ± 41	0	0	792.7 ± 21.8
Veneer_OW01	1183 ± 62	1521 ± 25	72	34	777.6 ± 18.4
Veneer_FPP2	1199 ± 52	1477 ± 44	28	50	812.7 ± 13.0
Veneer_FPW1	1235 ± 39	1492 ± 44	43	86	828.2 ± 14.7
Veneer_Z33	1197 ± 57	1496 ± 58	47	48	800.5 ± 13.3

**Table 2:** Surface roughness and contact angle of water on uncoated and coated samples.

Sample	Roughness	Contact Angle
	(µm)	(°)
CB_ref	22.6	124
CB_OW01_s-l	53.8	16
CB_FPP2_s-l	27.6	-
CB_FPW1	64.0	-
CB_Z33_s-l	37.0	-
Veneer_ref	6.6	103
Veneer_OW01_s-l	6.0	-
Veneer_FPP2_s-l	10.2	17
Veneer_FPW1_s-l	1.2	-
Veneer_Z33_s-l	7.4	13

**Table 3:** Sheet resistance and line quality for the screen-printed samples.

Sample	Sheet Resistance	Sheet Resistance	Line Width	Minimum Gap Width
	15 × 15 mm Squares	0.5 × 18 mm Lines	(500 µm Nominal)	
	(mΩ/sq)	(mΩ/sq)	(µm)	(µm)
CB_ref	38 ± 6	151 ± 11	1263	>500
CB_OW01	36 ± 21	347 ± n.a.	2080	>500
CB_FPP2	36 ± 9	274 ± 55	1975	>500
CB_FPW1	51 ± 9	402 ± 57	2067	>500
CB_Z33	39 ± 6	168 ± 56	2021	>500
Veneer_ref	91 ± 0	discontinuous	458	150
Veneer_OW01	163 ± 36	111 ± 22	400	300
Veneer_FPP2	113 ± 23	110 ± 12	441	300
Veneer_FPW1	104 ± 21	128 ± 12	420	200
Veneer_Z33	100 ± 18	71 ± n.a.	426	300

## Data Availability

More data available on request.
